# Early nutritional intervention can improve utilisation of vegetable-based diets in diploid and triploid Atlantic salmon (*Salmo salar* L.)

**DOI:** 10.1017/S0007114517001842

**Published:** 2017-07-24

**Authors:** Michael Clarkson, Herve Migaud, Christoforos Metochis, Luisa M. Vera, Daniel Leeming, Douglas R. Tocher, John F. Taylor

**Affiliations:** 1 Institute of Aquaculture, Faculty of Natural Sciences, University of Stirling, Stirling FK9 4LA, UK; 2 BioMar Ltd, North Shore Road, Grangemouth FK3 8UL, UK

**Keywords:** Atlantic salmon, Nutritional programming, Lipids, EPA: DHA, Vegetable raw material

## Abstract

The present study investigated nutritional programming in Atlantic salmon to improve utilisation of a vegetable-based diet. At first exogenous feeding, fry were fed either a marine-based diet (Diet M^stimulus^, 80% fishmeal (FM)/4% fish oil (FO)) or a vegetable-based diet (Diet V^stimulus^, 10% FM/0% FO) for 3 weeks. Subsequently, all fish were then fed under the same conditions with a commercial, marine-based, diet for 15 weeks and thereafter challenged with a second V diet (Diet V^challenge^, 10% FM/0% FO) for 6 weeks. Diploid and triploid siblings were run in parallel to examine ploidy effects. Growth performance, feed intake, nutrient utilisation and intestinal morphology were monitored. Fish initially given Diet V^stimulus^ (V-fish) showed 24 % higher growth rate and 23 % better feed efficiency compared with M-fish when later challenged with Diet V^challenge^. There was no difference in feed intake between nutritional histories, but increased nutrient retentions highlighted the improved utilisation of a V diet in V-fish. There were generally few significant effects of nutritional history or ploidy on enteritis scores in the distal intestine after the challenge phase as only V-triploids showed a significant increase (*P*<0·05) in total score. The data highlighted that the positive effects were most likely a result of nutritional programming and the ability to respond better when challenged later in life may be attributed to physiological and/or metabolic changes induced by the stimulus. This novel study showed the potential of nutritional programming to improve the use of plant raw material ingredients in feeds for Atlantic salmon.

Demand for farmed salmon heavily outweighs the availability of the raw materials, fishmeal (FM) and fish oil (FO), historically used to formulate feeds. According to the National Research Council^(^ ^1^ ^)^, the nutritional requirements for this carnivorous species during the freshwater stages include 42–50 % protein, containing essential amino acids, and 16–24 % lipid with an emphasis on long-chain (LC) *n*-3 fatty acids; EPA (20 : 5*n*-3) and DHA (22 : 6*n*-3) (0·5–1 %). Availability of these ingredients from marine resources is finite and alternative protein and lipid sources are required in order to sustain aquaculture development. Vegetable-derived proteins and oils are logical alternatives because of their high availability and relatively low production costs.

However, inclusion of plant ingredients in salmonid feeds can result in reduced feed utilisation. This may suggest a digestive and/or metabolic interference which can cause reduced growth performance and health issues. Reduced digestibility of plant ingredients in salmonid diets has been shown to correlate with reduced retention of protein and energy^(^ ^2^ ^–^ ^5^ ^)^, indicating lower metabolic activity and ultimately resulting in lower growth performance. Moreover, health implications such as distal intestinal (DI) enteritis, have been highlighted with some vegetable-based diets^(^ ^6^ ^–^ ^11^ ^)^. Several anti-nutritional factors (ANF) have been associated with detrimental effects on growth performance and health when using vegetable-based diets in aquafeeds^(^ ^12^ ^,^ ^13^ ^)^. Advances in feed technology have allowed further enrichment and refinement for several vegetable-based protein ingredients such as the processing of plant meals into protein concentrates, that is soya protein concentrate (SPC) by alcohol extraction, pea protein concentrate (PPC) by air classification, and wheat gluten (WG) by physical extraction^(^ ^13^ ^,^ ^14^ ^)^. These processes can reduce or remove ANF and ultimately reduce the associated health implications on gut morphology posed for salmonids^(^ ^7^ ^,^ ^14^ ^–^ ^17^ ^)^. Regardless, high inclusion of refined ingredients may still cause detriment to salmonids as seen in SPC^(^ ^18^ ^)^ and PPC^(^ ^10^ ^)^ but, at lower levels, such ingredients appear to be successful^(^ ^2^ ^,^ ^19^ ^–^ ^22^ ^)^. Moreover, blending reduced levels of SPC and faba bean protein concentrate previously demonstrated improved performance in salmon and reduced negative alterations to the gut transcriptome when compared with individual use of each ingredient^(^ ^23^ ^)^. Continuous refinement of alternative feeds is necessary to maximise benefits and minimise detrimental effects to the fish, with the aim to match the efficiency of traditional feeds optimally designed for carnivorous salmonids.

Nutritional programming has been considered as an option that may help to overcome problems associated with dietary replacement of FM and FO in aquafeeds. This concept involves nutritionally stimulating a physiological function during sensitive, early developmental stages, and has been shown to ‘programme’ or redirect particular metabolic processes in several different mammalian species^(^ ^24^ ^)^. The phenomenon has been investigated for several years with studies largely focused on rodents. Prenatal and postnatal investigations have concluded that a nutritional stimulus can trigger particular cellular development that can impact life development, for example growth performance^(^ ^25^ ^)^ and health^(^ ^26^ ^–^ ^28^ ^)^. The idea gained interest in human health studies, and animals such as primates^(^ ^29^ ^,^ ^30^ ^)^ and pigs^(^ ^31^ ^)^ have been used as models to understand lasting impacts of such nutritional interventions because of their similarities to human physiology. Typically, investigations have concluded that controlled prenatal or early postnatal nutrition can improve growth and development, and reduce incidence or severity of particular health issues such as obesity and CVD. With regards to agriculture, understanding the consequential importance of the impact of early nutrition will help to (i) improve production and (ii) mitigate potential problems. Evidence suggested that improved performance and increased parasitic resistance in sheep could result from nutritional interventions during the weaning period^(^ ^32^ ^)^. To date, there have been only a few similar studies in teleost species. A short exposure of a soyabean meal (SBM) diet at first feeding in rainbow trout (*Oncorhynchus mykiss*) improved the palatability and utilisation of the same diet later in life^(^ ^33^ ^)^. The programming theory has also been investigated in zebrafish (*Danio rerio*) and an early nutritional intervention has shown to alter carbohydrate digestion in later life^(^ ^34^ ^)^. Moreover, investigation of early programming on a molecular level appears to alter some physiological pathways involved in gut function in both zebrafish (*D. rerio*) and Gilthead seabream (*Sparus aurata*)^(^ ^35^ ^,^ ^36^ ^)^.

The overall objective of the present study was to determine if the concept of nutritional programming could operate in Atlantic salmon. The specific aims were, first, to determine whether the provision of Atlantic salmon fry with a vegetable-based diet at first exogenous feeding was able to physiologically adapt the fish to accept and more efficiently utilise the same diet at a later life stage without compromising growth performance and health. Second, given the growing interest in the use of triploid fish in aquaculture, and indications that growth performance and feed efficiency (FE) and, in turn, dietary requirements, may vary between triploid and diploid salmon^(^ ^37^ ^–^ ^40^ ^)^, the concept was tested in both diploid and triploid salmon in order to establish, not only if there were differences in their performance in response to such changes in raw materials, but also to determine if the concept of nutritional programming was affected by ploidy.

## Methods

The feeding trial was carried out at the University of Stirling temperate freshwater facilities with all experimental procedures conducted in compliance with the Animals Scientific Procedures Act 1986 (Home Office Code of Practice, HMSO, London, January 1997) under project licence PPL70/7916 ‘Environmental Regulation of Fish Physiology’ H. M.) in accordance with EU regulation (EC Directive 86/609/EEC). All experimentation performed at the Institute of Aquaculture (IoA) was subject to an ethical review process carried out by the University of Stirling Animal Welfare and Ethical Review Board before the work being approved.

### Experimental diets

Diets used in this study were formulated by BioMar UK Ltd and manufactured at the BioMar Tech Centre. Diet formulations and compositions are shown in Table 1. In brief, the marine stimulus diet (Diet M^stimulus^) was a formulation almost exclusively based on FM (80 % FM) as protein source and FO (4 % FO) as lipid source. The vegetable-based stimulus diet (Diet V^stimulus^) contained only a low proportion of FM (10 % FM) and a mixture of plant protein concentrates (SPC, PPC and WG) as protein sources, whereas rapeseed oil was the sole added lipid source (0 % FO). The vegetable-based challenge diet (Diet V^challenge^) contained the same FM/FO % and ingredients as Diet V^stimulus^, only a different composition to account for size of pellet.

**Table 1:** Formulation, proximate composition and fatty acid composition of the high marine diet (Diet M^stimulus^) and low fishmeal/fish oil diets (Diet V^stimulus^ and Diet V^challenge^) used in the respective feeding phases

Experimental phases...	Stimulus phase		Challenge phase
Diets...	M^stimulus^	V^stimulus^	V^challenge^
Ingredients (g/kg)			
Fishmeal*	648·4	50·0	50·0
Crustacean and fish peptones†	146·0	50·0	50·0
SPC‡	–	163·7	90·2
Wheat gluten§	–	214·0	181·7
PPC||	–	210·0	245·7
Wheat¶	135·9	139·9	134·4
Fish oil**	40·0	·	·
Rapeseed oil§	–	60·0	170·6
Vitamins and minerals††	22·7	54·8	52·5
Amino acids‡‡	7·0	57·6	25·0
Analysed proximate composition			
Lipid – crude (%)	13·3	11·3	21·6
Protein – crude (%)	57·1	56·6	49·6
Energy – gross (MJ/kg)	20·5	20·6	22·7
All fatty acids (% total fatty acids)			
PUFA	40·6	37·6	33·3
LA (18 : 2*n*-6)	4·8	25·8	22·9
ALA (18 : 3*n*-3)	1·3	8·2	8·9
EPA (20 : 5*n*-3)	13·0	1·4	0·6
DHA (22 : 6*n*-3)	12·1	1·4	0·6

SPC, soya protein concentrate; PPC, pea protein concentrate; LA, linoleic acid; ALA, *α*-linolenic acid.

* Feed Services Bremen.

† Aker BioMarine.

‡ Caramuru.

§ Cargill.

|| Agrident.

¶ WN Lindsay.

** ED&F Man.

†† DSM.

‡‡ Evonik.

### Fish stock and culture conditions

Eggs and milt from unrelated Atlantic salmon two sea-winter broodstock (Landcatch Natural Selection Ltd) were collected in December 2014 and transferred to the IoA (University of Stirling, Scotland) where the experiment took place in the temperate freshwater recirculation facility. Eggs were divided into two groups (approximately 1680 eggs each) for ploidy differentiation. Triploidy was induced in one group, with eggs subjected to 9500 psi (pounds per square inch) of hydrostatic pressure for 6·25 min at 8°C, 37 min post-fertilisation^(^ ^41^ ^)^. Both groups of eggs were incubated at a relatively low temperature of 5·6±0·1°C to account for the triploid salmon requiring a lower thermal regimen for optimal performance^(^ ^42^ ^)^. Towards the end of the alevin stage (approximately 950°d), fish were transferred to 12×0·3 m² tanks under 24 h light, and water temperature was increased over 11 d before first feeding and maintained until the end of the experiment at 12·7±0·5°C.

### Feeding trial

During the first 3 weeks of exogenous feeding, termed the ‘stimulus’ phase, diploid (2 N) and triploid (3 N) salmon were fed either Diet M^stimulus^ or Diet V^stimulus^ using automatic feeders (Arvo-Tec Feeding System) for two 4-h periods daily. Each of the four treatments (2NM, 3NM, 2NV, 3NV) were triplicated with 260 fish stocked per tank. During the ‘marine’ phase, all groups were fed a commercial marine-based diet (55 % protein and 20 % lipid, with blends containing FM and FO) for 15 weeks under the same conditions. The ‘challenge’ phase consisted of all groups then being fed Diet V^challenge^ for a further 6 weeks before the trial was concluded. Throughout the experiment, all groups of fish were fed to satiation plus 10 % excess and survival was monitored daily. The only time the fish in the present study were fed different diets was during the stimulus phase when fish were fed either Diet M^stimulus^ or Diet V^stimulus^. For simplicity, the terms ‘M-fish’ and ‘V-fish’ will be used, respectively.

### Verification of ploidy

To confirm ploidy status, red blood smears were prepared from samples taken from the caudal peduncle of euthanised fish (*n* 20/ploidy). Air dried slides were fixed in 100 % methanol and then placed into Giemsa stain for 10 min. Slides were digitised using a slide scanner at 20× magnification (Axio Scan.Z1; Zeiss) and erythrocyte length and diameter was determined by Fiji software (ImageJ). A total of thirty randomly chosen nuclei per slide were measured to the nearest 0·01 μm and a total mean taken for presumed diploid and triploid fish. Diploid groups had significantly smaller erythrocyte nuclear lengths, with no overlaps with the pressure shocked triploid groups (2 N, 7·4–8·5 μm; 3 N, 9·5–11·3 μm) confirming that all fish that were subjected to hydrostatic pressure shock were likely to be triploid.

### Sampling procedures

For growth assessment, following a 24-h fasting period, individuals (*n* 30/tank) were weighed (body weight, BW) at the relevant dietary transition periods from the initial (i) to the final (f) sampling point. Fish were sedated before weight measurement (Tricaine, 50 parts per million (ppm); Pharmaq). Growth rate was calculated using the thermal growth coefficient (TGC, % BW °C/d). For carcass and tissue analyses, fish were randomly selected and euthanised (Tricaine, 1000 ppm; Pharmaq).

### Feed intake

Feed intake (FI) was monitored during the final 16 d of the marine phase and for the duration of the challenge phase (41 d). All waste was siphoned out of the tank and uneaten feed was separated from faeces and any detritus. The uneaten pellets were weighed and converted to pellet number and dry weight from earlier calculations. FI was measured and revised for contrasting growth rates between populations; per 100 g average BW (% BW/d). The response of BW gain to FI during the challenge phase was measured as FE) and calculated as (BW_f_−BW_i_)/FI. Nutrient and energy utilisation efficiency was calculated using determined biochemical compositions (proximate analysis) of whole body fish and feeds with the influence on BW gain.

### Proximate composition

Proximate composition of feeds and 24-h starved whole fish were determined according to standard procedures^(^ ^43^ ^)^. Samples were collected at relevant transition periods after lethal anaesthesia (Tricaine, 1000 ppm) as described above. Whole fish were homogenised in a blender (Waring Laboratory Science) to produce pates, and feeds were ground before analyses. Moisture contents were obtained after drying in an oven at 110°C for 24 h and ash content determined after incineration at 600°C for 16 h. Crude protein content was measured by determining N content (N×6·25) using automated Kjeldahl analysis (Tecator Kjeltec Auto 1030 analyser; Foss) and crude lipid content was determined after acid hydrolysis followed by Soxhlet lipid extraction (Tecator Soxtec system 2050 Auto Extraction apparatus; Foss). Energy content was measured using bomb calorimetry calibrated with benzoic acid (Gallenkamp Autobomb; Gallenkamp & Co. Ltd).

### Fatty acid composition

Total lipid was extracted from diets, whole fish and tissue pates by homogenisation in chloroform/methanol (2:1, v/v)^(^ ^44^ ^)^. Fatty acid methyl esters (FAME) were prepared from total lipid by acid-catalysed transesterification at 50°C for 16 h^(^ ^45^ ^)^, and FAME extracted and purified as described previously^(^ ^46^ ^)^. FAME were separated and quantified by GLC using a Fisons GC-8160 (Thermo Scientific) equipped with a 30 m×0·32 mm internal diameter×0·25 μm ZB-wax column (Phenomenex), on-column injector and a flame ionisation detector. Data were collected and processed using Chromcard for Windows (version 2.01; Thermoquest Italia S.p.A.). Individual FAME were identified by comparison to known standards and published data^(^ ^46^ ^)^.

### Distal intestine histology

Randomly selected fish were euthanised (Tricaine, 1000 ppm) after a 24 h fasting period at the end of the marine (*n* 2/tank) and the challenge phase (*n* 5/tank). The entire DI was dissected and rinsed of faecal material in 4°C saline solution before storage in Serra fixative (ethanol–formalin–glacial acetic acid; 6:3:1) for 24 h and subsequently in ethanol (70 %). Samples were later processed according to standard histological methods. In brief, the samples were dehydrated in ethanol, equilibrated in xylene and embedded in paraffin. Longitudinal cuts (i.e. perpendicular to the macroscopically visible circular folds) of approximately 5 μm were stained with haematoxylin–eosin. The sections were examined by experienced personnel in three independent blinded evaluations. The following histological characteristics were evaluated according to a previous study^(^ ^47^ ^)^: width of the lamina propria (LP) and sub-epithelial mucosa (SEM), infiltration of SEM by eosinophilic granulocytes (EG), infiltration of the intra-epithelial spaces by lymphocytes (IEL) and the mitotic activity in mucosal fold base. Details of the histopathological scoring system utilised for the DI samples is given in Table 2.

**Table 2:** Description of scoring system covering a range of parameters used to assess severity of enteritis^(^ ^42^ ^)^

Scores	Parameter
LP	
1	Normal size LP
2	Normal to moderate size LP
3	Moderate size LP
4	Moderate to large size LP
5	Large size LP
EG	
1	Few in SEM
2	Increased number in SEM (multiple layers)
3	Increased number in SEM and migration into LP
4	Diffused number in LP and SEM
5	Dense EG in SEM and LP
SEM	
1	Normal SEM
2	Normal to moderate size SEM
3	Moderate size SEM
4	Moderate to large size SEM
5	Large size SEM
IEL	
1	Rare IEL (1/20 epithelial cells)
2	Mild (focal increase in numbers migrating towards the apical cytoplasm of epithelium; few scattered)
3	Moderate (increased numbers often towards the apical cytoplasm of epithelium)
4	Severe (marked increase in IEL)
Mucosal fold base mitotic activity	
1	Normal (2–3 mitotic epithelial cells)
2	Moderate (5–10 mitotic epithelial cells; some leucocytes might exhibit mitotic activity)
3	High (>10 mitotic epithelial and increment in intestinal leucocyte mitotic activity)

LP, lamina propria; EG, eosinophilic granulocytes; SEM, sub-epithelial mucosa; IEL, intra-epithelial lymphocytes.

### Statistical analysis

Minitab 17 Statistical Software (2010) was used for all statistical analyses. After confirming normality and homogeneity of variance in the data using the Kolmogorov–Smirnov test and Levene’s test, a two-way ANOVA was performed on independent parameters considering nutritional history (NH, Diet M^stimulus^ and Diet V^stimulus^), ploidy (2 N and 3 N), and their interaction. Percentage data were transformed using the arcsine square root function. Significance was accepted at *P*<0·05 and Tukey’s *post hoc* test was used to compare significantly different means. To investigate solely nutritional influence, differences shown within a given ploidy were analysed using a Kruskal–Wallis test. Histological scores were analysed statistically using a Kruskal–Wallis test followed by Dunn’s *post hoc* test for non-parametric, categorical comparison.

## Results

### Growth performance

Survival (%) was slightly lower in V-fish and triploids during both the stimulus and marine phases (Table 3). However, during the challenge phase, there were no effects of nutritional history or ploidy on survival. During the stimulus phase, growth rate as measured by TGC was higher in M-fish compared with V-fish, and in diploids compared with triploids (Table 3). There was also a significant difference in growth rate during the marine phase with M-fish showing higher TGC than V-fish, although this was only significant in diploids. In general, triploids significantly outgrew diploids (*P*<0·05) during the marine phase. These effects on growth were observed in the BW of the fish at the beginning and end of the FI trial carried out in the last 16 d of the marine phase, with both initial and final BW being higher in M-fish than V-fish, significantly so in both ploidies, and also significantly higher in triploids than in diploids (Table 4). In contrast, during the challenge phase, growth rate for both diploids and triploids was significantly greater in V-fish compared to M-fish as evidenced by the higher TGC values (Table 3). Growth of triploids was also significantly greater than that of diploids during the challenge phase. These growth differences were reflected in final BW so that, despite the fact that weights of M-fish were higher than those of V-fish at the beginning of the challenge phase, there were no significant differences in final BW of V-fish and M-fish at the end of the challenge phase (Table 5).

**Table 3:** Survival, growth rate and feed efficiency of fish during each of the three nutritional phases; stimulus, marine and challenge† (Mean values with their standard errors, *n* 3)

Ploidy...	Diploid				Triploid						
NH...	M^stimulus^		V^stimulus^		M^stimulus^		V^stimulus^		*P*		
	Mean	sem	Mean	sem	Mean	sem	Mean	sem	Ploidy	NH	Ploidy×NH
Survival (%)											
Stimulus phase	98·3	–	89·2	–	95·2		79·7		–	–	–
Marine phase	96·7	2·0	92·2	0·4	79·8	1·7	72·9	5·1	0·000	0·046	NS
Challenge phase	99·5	0·3	99·6*	0·3	98·3*	0·9	98·8*	0·2	NS	NS	NS
Growth rate (TGC, % BW °C/d)											
Stimulus phase	0·8	–	0·5	–	0·7		0·3		–	–	–
Marine phase	1·4^a^	0·0	1·3^b^	0·0	1·5	0·0	1·5	0·0	0·000	0·024	NS
Challenge phase	1·0^b^*	0·0	1·3^a^	0·1	1·1^b^*	0·1	1·5^a^	0·0	0·009	0·001	NS
Feed efficiency											
Stimulus phase	–	–	–	–	–	–	–	–	–	–	–
Marine phase	1·6^a^	0·1	1·2^b^	0·0	1·4	0·1	1·7	0·1	NS	NS	0·005
Challenge phase	1·2^b^*	0·1	1·6^a^*	0·0	1·2	0·0	1·5	0·0	NS	0·001	NS

NH, nutritional history; TGC, thermal growth coefficient; BW, body weight. ^a,b^ Significant differences between diets within a given ploidy. * Significant differences between phases within a given treatment.

† Based on their ploidy status (diploid or triploid) and their nutritional history during the stimulus phase (Diet M^stimulus^ or Diet V^stimulus^). Percentage data were arcsine transformed for statistical analysis. Significance was calculated between ploidy, NH and their interaction (Ploidy×NH), and was accepted at *P*<0·05.

**Table 4:** Growth parameters, feed intake (FI) and feed utilisation during the marine phase* (Mean values with their standard errors, *n* 3)

Ploidy...	Diploid				Triploid						
NH...	M^stimulus^		V^stimulus^		M^stimulus^		V^stimulus^		*P*		
	Mean	sem	Mean	sem	Mean	sem	Mean	sem	Ploidy	NH	Ploidy×NH
Growth parameters (×in/d)											
Initial body weight (g)	11·0^a^	0·2	9·8^b^	0·2	13·0^a^	0·3	10·9^b^	0·7	0·006	0·005	NS
Final body weight (g)	17·9^a^	0·0	14·2^b^	0·0	19·9^a^	1·2	16·9^b^	0·6	0·007	0·001	NS
Protein gain (g)	1·2^a^	0·0	0·8^b^	0·0	1·1	0·1	1·1	0·1	NS	0·029	0·043
Lipid gain (g)	1·0^a^	0·1	0·4^b^	0·1	0·9	0·2	0·5	0·0	NS	0·009	NS
Energy gain (kJ)	61·3^a^	3·8	39·7^b^	3·6	64·3	7·7	56·3	9·2	NS	0·046	NS
Voluntary FI											
FI (% BW/d)	1·9	0·1	1·9	0·1	1·9	0·0	1·9	0·0	NS	NS	NS
Nutrient and energy utilisation efficiency (% intake)											
Protein retention	50·3^a^	1·4	37·8^b^	1·1	41·5	3·2	49·9	3·0	NS	NS	0·003
Lipid retention	99·2^a^	9·7	54·0^b^	13·1	76·7	11·1	56·3	0·7	NS	0·022	NS
Energy retention	71·0	4·6	54·4	4·1	64·2	5·8	68·8	8·5	NS	NS	NS
EPA (20 : 5*n*-3) retention	22·6	1·7	17·1	1·7	20·2	4·2	12·7	6·0	NS	NS	NS
DHA (22 : 6*n*-3) retention	104·9^a^	5·8	78·9^b^	7·9	89·7	19·1	55·7	36·0	NS	NS	NS

NH, nutritional history; BW, body weight. ^a,b^ Significant differences within a given ploidy.

* Based on their ploidy status (diploid or triploid) and their nutritional history during the stimulus phase (Diet M^stimulus^ or Diet V^stimulus^). Percentage data were arcsine transformed for statistical analysis. Significance was calculated between ploidy, NH and their interaction (Ploidy×NH), and was accepted at *P*<0·05.

**Table 5:** Growth parameters, feed intake (FI) and feed utilisation during the challenge phase* (Mean values with their standard errors, *n* 3)

Ploidy...	Diploid				Triploid						
NH...	M^stimulus^		V^stimulus^		M^stimulus^		V^stimulus^		*P*		
	Mean	sem	Mean	sem	Mean	sem	Mean	sem	Ploidy	NH	Ploidy×NH
Growth parameters (×in/d)											
Initial body weight (g)	17·9^a^	0·0	14·2^b^	0·0	19·9^a^	1·2	16·9^b^	0·6	0·007	0·001	NS
Final body weight (g)	30·0	0·4	28·8	1·3	35·5	1·1	37·1	1·1	0·000	NS	NS
Protein gain (g)	2·1	0·2	2·3	0·2	2·7	0·1	3·1	0·0	0·003	NS	NS
Lipid gain (g)	1·1^b^	0·1	1·8^a^	0·3	2·2	0·2	2·7	0·2	0·004	0·029	NS
Energy gain (kJ)	105·7^b^	1·5	132·0^a^	14·3	148·5	10·6	176·3	9·1	0·005	0·040	NS
Voluntary FI											
FI (% BW/d)	1·0	0·1	1·0	0·1	1·2	0·1	1·2	0·0	0·044	NS	NS
Nutrient and energy utilisation efficiency (% intake)											
Protein retention	41·4^b^	3·5	53·9^a^	0·5	41·5	2·1	46·4	2·2	NS	0·011	NS
Lipid retention	50·4^b^	6·1	92·4^a^	7·2	72·8	4·0	87·6	3·2	NS	0·002	NS
Energy retention	46·1^b^	2·8	65·3^a^	2·0	48·5	1·4	56·6	0·1	NS	0·000	0·043
EPA (20 : 5*n*-3) retention	−8·0^b^	60·8	165·5^a^	18·9	−13·9	15·5	121·8	36·4	NS	0·006	NS
DHA (22 : 6*n*-3) retention	237·7^b^	152·1	797·0^a^	50·5	200·8	34·7	618·5	67·0	NS	0·002	NS

NH, nutritional history; BW, body weight. ^a,b^ Significant differences within a given ploidy.

* Based on their ploidy status (diploid or triploid) and their NH during the stimulus phase (Diet M^stimulus^ or Diet V^stimulus^). Percentage data were arcsine transformed for statistical analysis. Significance was calculated between ploidy, NH and their interaction (Ploidy×NH), and was accepted at *P*<0·05.

### Feed intake

When FI was corrected (% BW/d), no impacts of nutritional history or ploidy were observed between treatments during either feeding phase (Tables 4 and 5).

### Feed utilisation

During the marine phase FE was higher in M-fish compared with V-fish, significantly so with diploids (Table 3). This resulted in M-fish showing higher protein, lipid and energy gains and retentions in the marine phase compared with V-fish, again significant in diploids with triploids showing identical trends (Table 4). In contrast, the opposite effects on nutrient and energy utilisation were observed during the challenge phase. Thus, FE was higher in V-fish compared with M-fish, significantly so in diploids (Table 3). This resulted in V-fish showing higher protein, lipid and energy gains compared with M-fish, although only significant with lipid and energy gain in diploids. Consistent with these data, protein, lipid and energy retentions in the challenge phase were higher in V-fish than M-fish, significant in diploids with triploids showing identical trends (Table 5). The effects on nutrient utilisation efficiency/retention were therefore consistent across all nutrients, reflected in the fact that there was no effect of nutritional history on whole body proximate composition (Table 6).

**Table 6:** Whole fish proximate composition before and after the challenge period† (Mean values with their standard errors, *n* 3)

Ploidy...	Diploid				Triploid						
NH...	M^stimulus^		V^stimulus^		M^stimulus^		V^stimulus^		*P*		
	Mean	sem	Mean	sem	Mean	sem	Mean	sem	Ploidy	NH	Ploidy×NH
Pre-challenge phase											
DM (%)	28·9	0·2	28·3	0·4	28·6	0·0	28·4	0·5	NS	NS	NS
Lipid – crude (%)	10·2	0·5	9·9	0·3	10·6	0·1	10·1	0·6	NS	NS	NS
Protein – crude (%)	15·1	0·2	14·6	0·1	14·5	0·0	14·7	0·1	NS	NS	0·012
Ash (%)	2·3	0·1	2·2	0·0	2·0	0·0	2·2	0·1	NS	NS	NS
Energy – gross (kJ/100 g)	7·8	0·1	7·7	0·2	7·9	0·0	7·7	0·2	NS	NS	NS
Post-challenge phase											
DM (%)	30·1*	0·3	30·2*	0·4	31·0*	0·3	30·3*	0·1	NS	NS	NS
Lipid – crude (%)	10·9	0·3	11·8*	0·4	12·0	0·7	12·0*	0·2	NS	NS	NS
Protein – crude (%)	15·8	0·3	15·3*	0·0	15·7*	0·3	15·1	0·1	NS	0·031	NS
Ash (%)	2·2	0·1	2·2	0·1	2·1	0·1	2·2	0·1	NS	NS	NS
Energy – gross (kJ/100 g)	8·2	0·1	8·4*	0·1	8·6*	0·2	8·4*	0·1	NS	NS	NS

NH, nutritional history. Significant differences between diets within a given ploidy. * Significant differences between phases within a given treatment.

† Percentage data were arcsine transformed for statistical analysis.

### Fatty acid retention and composition

The same trend as observed for the macronutrients was also found in EPA and DHA retention in the marine and challenge phases, respectively (Tables 4 and 5). Importantly, M-fish lost EPA during the challenge phase, whereas V-fish positively retained EPA (Table 5). In contrast, all fish retained DHA during the challenge phase although retention was far greater in V-fish than in M-fish. Unsurprisingly, the reverse trend was seen during the marine phase, with M-fish have the greater EPA and DHA retentions. As with most parameters, the effects of nutritional history on EPA and DHA retentions were significant for diploids, with triploids showing identical but non-significant trends. Fatty acid profiles of whole body, liver, and pyloric caeca pre-challenge (end of marine phase) and post-challenge phase reflected the dietary fatty acid compositions of the commercial diet (fed in the pre-challenge phase) and Diet V^challenge^ (fed during the challenge phase) in all fish irrespective of nutritional history or ploidy (Table 7). Therefore, total SFA and *n*-3 PUFA (especially EPA and DHA) decreased, and total monoenes and *n*-6 PUFA (especially linoleic acid, 18 : 2*n*-6) increased in all tissues in all fish from pre- to post-challenge. The differences in EPA and DHA retention (based on absolute contents of whole body) between fish of different nutritional history were not reflected in the relative proportions of the fatty acids in whole body.

**Table 7:** Fatty acid compositions (% fatty acid methyl esters) of whole body, liver and pyloric caeca before and after the challenge phase† (Mean values and standard deviations, *n* 3)

	Pre-challenge phase								Post-challenge phase							
Ploidy...	Diploid				Triploid				Diploid				Triploid			
NH...	M^stimulus^		V^stimulus^		M^stimulus^		V^stimulus^		M^stimulus^		V^stimulus^		M^stimulus^		V^stimulus^	
	Mean	sd	Mean	sd	Mean	sd	Mean	sd	Mean	sd	Mean	sd	Mean	sd	Mean	sd
Whole body (%)																
Total saturated	25·3^a^	0·3	24·1^b^	0·3	25·0	1·1	24·2	0·8	19·1*	0·7	18·9*	0·6	18·7*	0·5	18·4*	0·2
Total monoenes	46·5	0·6	47·1	0·6	46·8	0·8	46·7	0·5	50·9*	0·6	51·2*	0·6	52·1*	0·6	52·1*	0·1
18 : 2*n*-6	4·7	0·1	5·0	0·2	4·7^b^	0·1	4·9^a^	0·1	11·4*	0·7	11·5*	0·7	11·9*	0·6	11·9*	0·3
20 : 4*n*-6	0·5	0·0	0·5	0·0	0·5	0·0	0·5	0·0	0·7*	0·0	0·7*	0·0	0·7*	0·0	0·7*	0·0
Total *n*-6 PUFA	6·3	0·1	6·7	0·2	6·3	0·2	6·6	0·2	14·7*	0·8	14·8*	0·9	15·4*	0·7	15·3*	0·5
18 : 3*n*-3	0·9	0·0	1·1	0·1	0·9^b^	0·0	1·0^a^	0·0	3·0*	0·2	3·00*	0·2	3·1*	0·2	3·2*	0·1
20 : 5*n*-3	4·0	0·0	4·0	0·2	4·1	0·2	4·2	0·0	2·0*	0·2	2·0*	0·2	1·7*	0·2	1·8*	0·1
22 : 6*n*-3	12·4	0·1	12·4	0·6	12·2	0·4	12·6	0·4	6·8*	0·5	6·7*	0·7	5·7*	0·6	6·0*	0·4
Total *n*-3 PUFA	21·0	0·1	21·2	0·8	21·1	0·7	21·6	0·4	14·8*	0·7	14·6*	0·9	13·4*	0·7	13·8*	0·6
Total PUFA	28·3	0·3	28·8	0·9	28·2	0·9	29·1	0·6	30·0*	0·3	29·9	0·3	29·2	0·1	29·5	0·1
Liver (%)																
Total saturated	26·8	0·6	26·6	1·5	26·6	0·5	26·3	1·2	16·2*	1·1	17·2*	1·2	18·3*	1·1	17·7*	1·6
Total monoenes	27·5	1·1	29·7	5·2	27·8	3·5	29·5	1·9	50·8*	2·1	50·8*	3·0	47·8*	2·4	50·3*	4·8
18 : 2*n*-6	2·4	0·1	2·4	0·2	2·4	0·3	2·4	0·1	9·3*	0·9	9·0*	0·4	8·2*	0·7	9·0*	0·8
20 : 4*n*-6	3·1	0·2	2·9	0·4	2·9	0·3	2·9	0·2	4·6*	0·8	4·3*	0·4	5·2*	0·9	4·7*	1·1
Total *n*-6 PUFA	7·2	0·1	7·0	0·2	7·2	0·1	7·1	0·2	19·6*	0·9	19·4*	0·0	19·2*	0·5	19·4*	0·9
18 : 3*n*-3	0·3	0·0	0·3	0·0	0·3^b^	0·1	0·3^a^	0·0	1·2*	0·2	1·2*	0·0	1·1*	0·1	1·2*	0·1
20 : 5*n*-3	5·9	0·2	5·8	0·4	6·0	0·2	6·0	0·1	1·4*	0·2	1·4*	0·3	1·7*	0·3	1·5*	0·3
22 : 6*n*-3	30·3	1·4	28·8	3·5	29·9	3·3	28·8	1·2	9·5*	0·6	8·8*	1·4	10·5*	1·2	8·7*	2·0
Total *n*-3 PUFA	38·4	1·0	36·7	3·8	38·3	3·3	37·1	1·1	13·3*	0·7	12·7*	1·8	14·6*	1·4	12·6*	2·3
Total PUFA	45·7	1·1	43·7	3·9	45·6	3·2	44·2	1·1	32·9*	1·5	32·1*	1·8	33·9*	1·3	32·1*	3·2
Pyloric caeca (%)																
Total saturated	27·9	0·7	27·2	0·4	27·7	0·5	27·7	0·5	19·1*	0·5	18·2*	0·4	17·8*	0·8	17·8*	0·4
Total monoenes	45·3	0·1	44·1	0·9	43·2	0·2	42·5	2·7	50·0*	0·8	50·2*	0·3	52·0^a^*	0·5	50·0^b^*	0·7
18 : 2*n*-6	4·6	0·2	4·6	0·2	4·6	0·2	4·5	0·1	11·3^b^*	0·3	12·1^a^*	0·3	12·3*	0·5	12·6*	0·9
20 : 4*n*-6	0·6	0·0	0·6	0·0	0·7	0·0	0·8	0·2	1·1*	0·1	1·2*	0·1	0·9*	0·1	1·2	0·2
Total *n*-6 PUFA	6·2	0·2	6·4	0·2	6·4	0·2	6·4	0·3	15·5^b^*	0·3	16·9^a^*	0·6	16·5*	0·7	17·4*	1·5
18 : 3*n*-3	0·9	0·0	0·9	0·0	0·9	0·0	0·9	0·0	2·7^b^*	0·1	3·0^a^*	0·0	3·1*	0·2	3·1*	0·3
20 : 5*n*-3	3·4	0·3	3·5	0·2	3·5	0·1	3·5	0·2	1·8*	0·1	1·5*	0·2	1·5*	0·1	1·5*	0·4
22 : 6*n*-3	12·3^b^	0·3	13·8^a^	0·6	14·2	0·5	15·3	2·3	7·6*	0·6	7·2*	0·4	5·9*	0·2	7·2*	0·8
Total *n*-3 PUFA	19·8^b^	0·5	21·5^a^	0·7	21·8	0·5	22·7	2·6	15·0*	0·4	14·4*	0·9	13·3*	0·3	14·4*	1·0
Total PUFA	26·8^b^	0·7	28·7^a^	0·8	29·0	0·4	29·9	2·9	30·9*	0·4	31·6*	0·6	30·1^b^*	0·5	32·2^a^	0·7

NH, nutritional history. ^a,b^ Significant differences between diets within a given ploidy. * Significant differences between phases within a given treatment.

† Based on their ploidy status (diploid or triploid) and their NH during the stimulus phase (Diet M^stimulus^ or Diet V^stimulus^). Percentage data were arcsine transformed for statistical analysis. Significance was calculated between NH within each ploidy and was accepted at *P*<0·05.

### Distal intestine histology

Generally, histological assessment of DI at the end of the marine phase (pre-challenge) indicated that total enteritis scores were low and comparable across the four treatment groups (Table 8). No differences were found in the parameters analysed except for SEM, where V-diploids showed increased size compared with M-diploids, and lastly for IEL, where M-triploids showed significantly higher prevalence than V-triploids. Similarly, there were generally few significant effects of nutritional history or ploidy on enteritis scores in the distal intestine post-challenge (Table 8). However, in diploids, significantly higher SEM in V-fish compared with M-fish, as well as significantly higher total scoring of combined histological characteristics between these groups, was observed post-challenge phase. There were differences in scores within a given treatment between the pre- and post-challenge phases with all overall scores tending to be higher, although only significantly in V-triploids. Furthermore, EG scoring was significantly higher post-challenge regardless of nutritional history or ploidy. LP scoring was generally higher post-challenge, but only significantly so in M-triploids, and SEM was significantly higher in triploids post-challenge.

**Table 8:** Total scores and individual scores before and after the challenge phase for the different parameters used to determine severity of enteritis^(^ ^42^ ^)^ †(Mean values with their standard errors, *n* 3)

Ploidy...	Diploid				Triploid						
NH...	M^stimulus^		V^stimulus^		M^stimulus^		V^stimulus^		*P*		
	Mean	sem	Mean	sem	Mean	sem	Mean	sem	Ploidy	NH	Ploidy×NH
Pre-challenge phase											
LP	1·2	0·2	1·8	0·6	1·4	0·3	1·3	0·3	NS	NS	0·027
EG	1·1	0·1	1·2	0·3	1·1	0·2	1·1	0·2	NS	NS	NS
SEM	1·1^b^	0·3	1·4^a^	0·2	1·2	0·2	1·3	0·4	NS	NS	NS
IEL	2·0	0·4	2·0	0·4	2·1^a^	0·2	1·7^b^	0·3	NS	0·017	NS
MFBMA	1·1	0·2	1·4	0·2	1·2	0·1	1·4	0·2	NS	NS	NS
Total	6·5	0·9	7·8	1·4	7·1	0·4	6·8	1·1	NS	NS	NS
Post-challenge phase											
LP	1·6*	0·5	1·8	0·2	1·8	0·5	1·6	0·4	NS	NS	NS
EG	1·3*	0·2	1·5*	0·3	1·5*	0·3	1·5*	0·3	NS	NS	NS
SEM	1·3^b^	0·3	1·7^a^	0·2	1·8*	0·3	1·8	0·3	0·001	0·007	0·034
IEL	1·7	0·5	2·1	0·5	2·0	0·5	2·0	0·4	NS	NS	NS
MFBMA	1·3	0·3	1·5	0·3	1·4	0·4	1·5	0·3	NS	NS	NS
Total	7·2^b^	1·2	8·5^a^	0·8	8·5	1·6	8·4*	0·9	NS	NS	NS

NH, nutritional history; LP, lamina propria; EG, eosinophilic granulocytes; SEM, sub-epithelial mucosa; IEL, intra-epithelial lymphocytes; MFBMA, mucosal fold base mitotic activity. ^a,b^ Significant differences between diets within a given ploidy. * Significant differences between phases within a given treatment.

† Based on their ploidy status (diploid or triploid) and their NH during the stimulus phase (Diet M^stimulus^ or Diet V^stimulus^). Significance was calculated between ploidy, NH and their interaction (Ploidy×NH), and was accepted at *P*<0·05.

## Discussion

The present study confirmed that a short exposure to a vegetable-based diet (Diet V^stimulus^) during first exogenous feeding prepared or adapted Atlantic salmon, irrespective of ploidy, to better utilise a similar diet (Diet V^challenge^) when challenged later in life. This result is consistent with a previous trial which demonstrated a physiological adaptation in rainbow trout (*O. mykiss*) through nutritional programming^(^ ^33^ ^)^. In addition, the present study showed that while this ‘nutritional programming’ effect was highly significant in diploid salmon, the effect was also clearly apparent in triploid salmon albeit the greater variation in the triploid data often reduced the significance of these responses.

Throughout the majority of the trial, there was no significant difference in survival rates between nutritional histories within each ploidy. However, a difference was detected in the stimulus phase. V-fish showed a lower survival rate irrespective of ploidy. The same trend was found for growth during the stimulus phase as V-fish showed a lower growth rate. Reluctance to feed on vegetable-based diets is well documented in salmon and other species, especially when first presented^(^ ^48^ ^–^ ^50^ ^)^ and so it is likely that the greater mortality and lower growth in V-fish was initially because of the poor acceptance of Diet V^stimulus^ leading to reduced FI, although this could not be accurately measured in the first feeding fry in the present study. Failure or impaired establishment of first feeding of Diet V^stimulus^ may be explained by the physiological characteristics of a carnivorous teleost. Typically, Atlantic salmon develops anatomically according to the type and level of nutrients present in the maternal egg reserves^(^ ^51^ ^)^. This has been reviewed in several species concerning important organismal systems including reproductive structure and performance^(^ ^52^ ^)^. With regards to the gastrointestinal tract (GIT), morphological and functional differentiation occurs in the very early life stages and is influenced by environment and nutrition^(^ ^53^ ^)^. Overall, triploids had a lower survival rate during the stimulus and marine phases, which was consistent with previous studies^(^ ^54^ ^–^ ^56^ ^)^. Similarly, lower growth rates in triploid Atlantic salmon fry during the stimulus phase was also consistent with previous studies^(^ ^57^ ^,^ ^58^ ^)^. The experimental diets used in the present study were generally based on formulations for diploid salmon, and recent studies have indicated that triploid Atlantic salmon may have different nutritional requirements for certain physiological development, principally during the freshwater stage^(^ ^37^ ^,^ ^38^ ^,^ ^59^ ^,^ ^60^ ^)^. One may speculate that the increased proportions of vegetable-based proteins and oils currently used in commercial salmon feeds may have reduced nutrient bioavailability compared with FM/FO diets, which could perhaps highlight dietary deficiencies for triploids. However, high-quality protein concentrates have shown to be similar or more digestible than FM^(^ ^61^ ^,^ ^62^ ^)^. The reduced performance and survival of triploid fry during the stimulus phase may be explained by missing micronutrients in the formulation^(^ ^63^ ^,^ ^64^ ^)^. Also, this drawback may not be solely consequent of nutritional deficiency, but ploidy itself should be considered as a factor.

At the end of the stimulus phase V-fish were about 30 % smaller than M-fish, which was a potential confounding factor during the rest of the trial as fish size itself affects subsequent fish performance. This was taken fully into account in the present study. Ideally, therefore, the stimulus phase should not induce any major phenotypic changes. Very recent data on early nutritional stimuli suggested that 3 d may be sufficient to prompt a physiological adaptation to diet in zebrafish (*D. rerio*)^(^ ^35^ ^)^, although species-specific variation should not be excluded. During the marine phase, M-diploids had a higher growth rate than V-diploids. As discussed above, the difference in BW at the beginning of this phase could have had some impact on growth rate, but another factor could be potentially reduced acceptability of the commercial diet in V-fish. An investigation of nutritional programming in rainbow trout (*O. mykiss*) concluded that early nutritional intervention can alter transcriptional and physiological characteristics of the olfactory and gustatory systems to suit specific feed formulations^(^ ^33^ ^,^ ^65^ ^)^. Therefore, in this respect we can speculate that M-fish were already adapted for a commercial diet, whereas V-fish would have required time to adapt to it. FI was not measured at this point but was determined at the end of the marine phase when no difference between M-fish and V-fish was observed.

There was no difference in survival rate between fish during the challenge phase, but V-fish demonstrated significantly higher growth rates than M-fish. The switch in performance in response to Diet V^challenge^ had a positive effect that appeared to be related to the initial dietary stimulus. V-fish adapted to Diet V^challenge^ better than M-fish suggesting there was a degree of memory to the dietary stimulus. It has been previously described that environmental triggers can influence ‘multidimensional plasticity’ in different organisms, and suggested that transcriptional and physiological changes during early development could significantly affect the resilience of these organisms to different stressors^(^ ^66^ ^)^. The weights of V-fish at the end of the challenge phase were not higher than those of M-fish, because of the fact that V-fish were initially smaller due to lower growth during the stimulus and marine phases. However, the weights were comparable between nutritional histories, confirming the greater weight gain and higher growth rate (TGC) in V-fish during the challenge. The higher growth performance was reflected in greater protein, lipid, and energy gains of V-fish compared with M-fish during the challenge phase. Although these differences were generally not statistically significant, with the exception of lipid and energy gain in diploids, every nutrient group showed higher values in V-fish. Moreover, there was a ploidy effect as triploids appeared to gain significantly more macronutrients than diploids which has previously been reported in increased lipid uptake^(^ ^67^ ^)^. This may indicate physiological and metabolic differences between ploidy, with increased storage capacity as a consequence of larger cells being hypothesised^(^ ^68^ ^)^. An earlier study suggested that morphological differences in the GIT, predominantly caused by a reduction in intestinal cell numbers, could hinder the gut’s absorptive capability and therefore the intestinal efficiency of nutrient absorption in triploid salmon compared with diploid siblings^(^ ^69^ ^)^. Therefore, the data in the present study were not consistent with this hypothesis and highlighted the need for further research into the effects of ploidy on GIT morphology, physiology, enzymatic activities and intestinal nutrient absorption.

Because of the lower weight of V-fish, FI measurements during the marine and challenge phases were normalised to enable comparisons between groups, to account for the effect of fish weight on consumption rates. Previously, palatability has been an issue in salmonids fed with feeds containing low levels of marine and high amounts of plant-derived ingredients as fish have shown reluctance to consume these diets. Lower voluntary FI was observed in a previous study^(^ ^2^ ^)^ when Atlantic salmon were fed a SBM diet compared with a FM diet during the initial stages of the trial, although consumption rates were comparable towards the end of the trial suggesting that the fish finally accepted the diet possibly reflecting an adaptation to it. In the present study, there was no difference in FI irrespective of nutritional history or ploidy either at the end of the marine phase or during the challenge phase. However, there was a significant effect of nutritional history on FE, with V-fish having higher efficiency. Thus, the superior growth performance of V-fish during the challenge phase was likely the result of improved dietary nutrient utilisation. Furthermore, FE also confirmed that nutritional history had opposite effects during the marine and challenge phases, as previously discussed for growth performance. Thus, during the marine phase, M-fish exhibited higher FE compared with V-fish, consistent with the better growth of the M-fish during this phase, and the reverse trend was observed throughout the challenge. The FE data confirmed that the initial, brief exposure of salmon fry to Diet V^stimulus^ had a positive impact on these fish when they were challenged with Diet V^challenge^, and further suggested that physiological and metabolic changes and adaptations in these fish were, at least partly, responsible for the observed improvement in the utilisation of Diet V^challenge^ by V-fish. In contrast, a higher FI in rainbow trout was observed when re-introduced to a vegetable-based diet after an earlier nutritional stimulus^(^ ^33^ ^)^, suggesting that exposure to the diet early in life reduced the aversion of the fish to the challenge diet later in life. However, the trout initially fed the vegetable-based diet also showed improved FE when challenged with this diet later in life. The slight difference in the results between the earlier trout study and the present salmon study in terms of palatability may be related to differences in the dietary formulations used. For example, in the trout study the vegetable diet was completely devoid of marine ingredients (0 % FM and 0 % FO) and this may have posed an even more extreme challenge specifically in terms of palatability, but species-specific response should not be excluded. This highlighted the importance of optimising dietary formulations based on both taste preference and utilisation efficiency.

Retention of nutrients and energy was higher in diploid V-fish during the challenge phase which agreed with data in the earlier trial in trout mentioned above^(^ ^33^ ^)^. As with other parameters, this was the reverse trend from the previous marine phase which showed M-fish having better retentions than V-fish. Although an identical trend for all nutrients was observed in triploids, no significant differences between M- and V-triploid salmon were observed in either of the two phases. The present findings, however, could reflect the limited existing knowledge on the precise nutritional requirements of triploid salmon, which have been shown in previous studies to be higher than in diploids specifically in relation to phosphorus^(^ ^37^ ^,^ ^59^ ^)^ and histidine requirements^(^ ^38^ ^,^ ^60^ ^)^. V-fish positively retained EPA (20 : 5*n*-3) during the challenge phase whereas M-fish showed negative retention and, although DHA (22 : 6*n*-3) retention was positive in all fish irrespective of nutritional history or ploidy, retention was considerably greater in V-fish. This trend was found within both ploidies, but only significantly in diploids. Retention of DHA was consistently greater than that of EPA in all treatments, which is in accordance with previous reports suggesting selective catabolism of EPA over DHA in addition to a possible production of the fatty acids by endogenous biosynthesis pathways^(^ ^70^ ^,^ ^71^ ^)^. Thus, when considering the EPA and DHA retention data, any value that exceeds 100 % will include a proportion of the fatty acids that were biosynthesised from *α*-linolenic acid (ALA) (18 : 3*n*-3). Net production of DHA has previously been reported when Atlantic salmon^(^ ^72^ ^)^ and rainbow trout^(^ ^73^ ^)^ were fed with high inclusion of vegetable oil, and therefore low levels of dietary EPA and DHA. In the present study, net production of EPA was found in V-fish during the challenge period. This result is consistent with other studies^(^ ^73^ ^,^ ^74^ ^)^ where salmonids had received similarly low levels of dietary EPA and DHA (0·7 % of total FA). The selective retention of DHA over EPA, possibly tissue (i.e. neural tissue) specific, has been reported previously in several fish species^(^ ^75^ ^–^ ^77^ ^)^. DHA has a greater physiological importance in cell membrane composition and function when compared with EPA and therefore a higher valued essential fatty acid. The greater retention of both EPA and DHA in V-fish found in this study may reflect one possible and obvious metabolic adaptation in V-fish. It is likely that there is increased LC-PUFA biosynthesis through up-regulation of fatty acyl desaturase and elongase activities^(^ ^76^ ^,^ ^78^ ^)^. In an earlier trial in sea bass (*Dicentrarchus labrax*)^(^ ^79^ ^)^, expression of Δ-6 desaturase (*Δ6D* or *fads2d6*) was up-regulated in juveniles that had been previously fed an *n*-3 LC-PUFA-deficient diet as larvae. The Δ6D enzyme is the reported rate-limiting step in the conversion of ALA to EPA^(^ ^80^ ^)^. However, the early nutritional stimulus had no major effect on the final fatty acid compositions of either whole body or tissues in the present study. Perhaps as expected, fish and tissue fatty acid profiles at the end of the challenge phase reflected the dietary fatty acid compositions and therefore showed increased percentages of plant-derived C_18_ fatty acids (18 : 1*n*-9, 18 : 2*n*-6 and 18 : 3*n*-3) and decreased proportions *n*-3 LC-PUFA (EPA and DHA) in all treatment groups with no major influence of nutritional history (or ploidy)^(^ ^81^ ^)^. This change in tissue fatty acid profiles is in accordance with many studies on FM and FO substitution with alternative vegetable-based diets in salmon^(^ ^71^ ^,^ ^82^ ^–^ ^86^ ^)^. It is well established that, whereas up-regulation of LC-PUFA biosynthesis through increased expression and activity of fatty acid desaturase activities is a consistent response to vegetable diets in salmon, it is not sufficient to fully compensate for the lack of dietary EPA and DHA^(^ ^81^ ^,^ ^87^ ^)^. However, the challenge phase was 6 weeks, which is relatively short compared with most earlier studies on the effects of FO replacement with vegetable oils, and the differences in EPA and DHA retention between V- and M-fish recorded in the present study are large, and so it would be interesting to confirm if these would translate into higher levels of these key LC-PUFA in V-fish after longer feeding.

Overall, there were few notable differences in intestinal morphology before and after the challenge phase. Many of the earlier incidences of morphological changes in the gut and enteritis in Atlantic salmon were specifically related with the use of SBM in the diet^(^ ^8^ ^–^ ^9^ ^,^ ^88^ ^)^. Aqueous alcohol extraction of proteins from soyabeans or soya flour for the manufacturing of SPC, as used in the present study, reduces contents of specific ANF including saponins, lectins and soy-antigens^(^ ^14^ ^,^ ^16^ ^)^, which have been shown to be implicated in intestinal inflammation in salmon fed SBM^(^ ^12^ ^,^ ^89^ ^)^. Still, a significant difference in inflammation was found in V-diploids compared with M-diploids after the challenge phase. In a similar study investigating nutritional programming in zebrafish (*D. rerio*)^(^ ^35^ ^)^, fish previously exposed to SPC were shown to be more prone to intestinal inflammation when refed SPC than groups that had never been exposed to it. Although further processing of diets seem to be reducing incidence of enteritis, the present result suggests that further refinements are needed to equal the responses to current diets. The only treatment that showed an increase in the total scoring of intestinal integrity from pre- to post-challenge analysis was V-triploids. This could suggest that triploid salmon may be more sensitive to vegetable-based diets than their diploid counterparts and again highlighted the lack of knowledge regarding effects of ploidy on salmon morphological and physiological responses.

### Conclusions

The present study has indicated that nutritional programming may help to improve utilisation of a diet and reduce potential negative impacts associated with the use of alternative raw materials in aquafeeds. In particular, the present study has successfully demonstrated for the first time that Atlantic salmon can be adapted to utilise a vegetable-based diet more efficiently after an early nutritional intervention. Further optimisation of an effective stimulus both in terms of diet formulation and duration may further unlock the potential of this strategy. Importantly, the potential of salmon to apparently be programmed to be net producers of EPA and DHA should be further investigated. Biosynthesis of these health-promoting *n*-3 fatty acids shows promise when considering the limitation of raw material sources. Several metabolic pathways and key biochemical and physiological regulators have shown to be influenced in response to consumption of vegetable-based diets in Atlantic salmon^(^ ^90^ ^,^ ^91^ ^)^. Therefore, further studies are in progress to determine the molecular mechanisms potentially involved in this physiological adaptation.

## References

[1] National Research Council (2011) Nutrient Requirements of Fish and Shrimp. Washington, DC: National Academies Press.

[2] Refstie S, Storebakken T & Roem AJ (1998) Feed consumption and conversion in Atlantic salmon (*Salmo salar*) fed diets with fish meal, extracted soybean meal with reduced content of oligosaccharides, trypsin inhibitors, lectins and soya antigens. Aquaculture 162, 301–312.

[3] Refstie S, Korsøen ØJ, Storebakken T, et al (2000) Differing nutritional responses to dietary soybean meal in rainbow trout (*Oncorhynchus mykiss*) and Atlantic salmon (*Salmo salar*). Aquaculture 190, 49–63.

[4] Aslaksen MA, Kraugerud OF, Penn M, et al (2007) Screening of nutrient digestibilities and intestinal pathologies in Atlantic salmon, Salmo salar, fed diets with legumes, oilseeds or cereals. Aquaculture 272, 541–555.

[5] Wacyk J, Powell M, Rodnick K, et al (2012) Dietary protein source significantly alters growth performance, plasma variables and hepatic gene expression in rainbow trout (*Oncorhynchus mykiss*) fed amino acid balanced diets. Aquaculture 356-357, 223–234.

[6] Bæverfjord G & Krogdahl Å (1996) Development and regression of soybean meal induced enteritis in Atlantic salmon, *Salmo salar* L., distal intestine: a comparison with the intestines of fasted fish. J Fish Dis 19, 375–387.

[7] van den Ingh TSGAM, Olli JJ & Krogdahl Å (1996) Alcohol-soluble components in soybeans cause morphological changes in the distal intestine of Atlantic salmon, *Salmo salar* L. J Fish Dis 19, 47–53.

[8] Knudsen D, Jutfelt F, Sundh H, et al (2008) Dietary soya saponins increase gut permeability and play a key role in the onset of soyabean-induced enteritis in Atlantic salmon (*Salmo salar* L.). Br J Nutr 100, 120–129. 18167174 10.1017/S0007114507886338

[9] Urán PA, Scharama JW, Jaafari S, et al (2009) Variation in commercial sources of soybean meal influences the severity of enteritis in Atlantic salmon (*Salmo salar* L.). Aquacult Nutr 15, 492–499.

[10] Penn MH, Bendiksen EÅ, Campbell P, et al (2011) High level of dietary pea protein concentrate induces enteropathy in Atlantic salmon (*Salmo salar* L.). Aquaculture 310, 267–273.

[11] De Santis C, Ruohonen K, Tocher DR, et al (2015) Atlantic salmon (*Salmo salar*) parr as a model to predict the optimum inclusion of air classified faba bean protein concentrate in feeds for seawater salmon. Aquaculture 444, 70–78.

[12] Francis G, Makkar HPS & Becker K (2001) Antinutritional factors present in plant-derived alternate fish feed ingredients and their effects in fish. Aquaculture 199, 197–227.

[13] Gatlin DM, Barrows FT, Brown P, et al (2007) Expanding the utilisation of sustainable plant products in aquafeeds: a review. Aquacult Res 38, 551–579.

[14] Drew MD, Borgeson TL & Thiessen DL (2007) A review of processing of feed ingredients to enhance diet digestibility in finfish. Anim Feed Sci Technol 138, 118–136.

[15] Rumsey GL, Hughes SG & Winfree RA (1993) Chemical and nutritional evaluation of soya protein preparations as primary nitrogen sources for rainbow trout (*Oncorhynchus mykiss*). Anim Feed Sci Technol 40, 135–151.

[16] Bureau DP, Harris AM & Cho CY (1998) The effects of purified alcohol extracts from soy products on feed intake and growth of Chinook salmon (*Oncorhynchus tshawytscha*) and rainbow trout (*Oncorhynchus mykiss*). Aquaculture 161, 27–43.

[17] Burel C, Boujard T, Tulli F, et al (2000) Digestibility of extruded peas, extruded lupin, and rapeseed meal in rainbow trout (*Oncorhynchus mykiss*) and turbot (*Psetta maxima*). Aquaculture 188, 285–298.

[18] Mambrini M, Roem AJ, Cravédi JP, et al (1999) Effects of replacing fish meal with soy protein concentrate and of DL-methionine supplementation in high-energy, extruded diets on the growth and nutrient utilisation of rainbow trout, *Oncorhynchus mykiss* . J Anim Sci 77, 2990–2999. 10568469 10.2527/1999.77112990x

[19] Refstie S, Storebakken T, Bæverfjord G, et al (2001) Long-term protein and lipid growth of Atlantic salmon (*Salmo salar*) fed diets with partial replacement of fish meal by soy protein products at medium or high lipid level. Aquaculture 193, 91–106.

[20] Carter CG & Hauler RC (2000) Fish meal replacement by plant meals in extruded feeds for Atlantic salmon, *Salmo salar* L. Aquaculture 185, 299–311.

[21] Thiessen DL, Campbell GL & Adelizi PD (2003) Digestibility and growth performance of juvenile rainbow trout (*Oncorhynchus mykiss*) fed with pea and canola products. Aquacult Nutr 9, 67–75.

[22] Øverland M, Sørensen M, Storebakken T, et al (2009) Pea protein concentrate substituting fish meal or soybean meal in diets for Atlantic salmon (*Salmo salar*) – effect on growth performance, nutrient digestibility, carcass composition, gut health, and physical feed quality. Aquaculture 288, 305–311.

[23] Król E, Douglas A, Tocher DR, et al (2016) Differential responses of the gut transcriptome to plant protein diets in farmed Atlantic salmon. BMC Genomics 17, 156. 26925977 10.1186/s12864-016-2473-0 PMC4772681

[24] Lucas A (1998) Programming by nutrition: an experimental approach. J Nutr 128, 401S–406S. 9478036 10.1093/jn/128.2.401S

[25] Daenzer M, Ortmann S, Klaus S, et al (2002) Prenatal high protein exposure decreases energy expenditure and increases adiposity in young rats. J Nutr 132, 142–144. 11823569 10.1093/jn/132.2.142

[26] Heywood WE, Mian N, Milla PJ, et al (2004) Programming of defective rat pancreatic β-cell function in offspring from mothers fed a low-protein diet during gestation and the suckling periods. Clin Sci (Lond) 107, 37–45. 14982492 10.1042/CS20030350

[27] Khan IY, Taylor PD, Dekou V, et al (2003) Gender-linked hypertension in offspring of lard-fed pregnant rats. Hypertension 41, 168–175. 12511548 10.1161/01.hyp.0000047511.97879.fc

[28] Khan IY, Dekou V, Douglas G, et al (2005) A high-fat diet during rat pregnancy or suckling induces cardiovascular dysfunction in adult offspring. Am J Physiol 288, R127–R133. 10.1152/ajpregu.00354.2004 15308487

[29] Mott GE, Jackson EM, DeLallo L, et al (1995) Differences in cholesterol metabolism in juvenile baboons are programmed by breast- versus formula-feeding. J Lipid Res 36, 299–307. 7751817

[30] Mott GE & Lewis DS (2009) Baboon model for infant nutrition In The Baboon in Biomedical Research, pp. 255–264 [JL Van de Berg, S Williams-Blangero and SD Tardif, editors]. New York: Springer.

[31] Guilloteau P, Zabielski R, Hammon HM, et al (2010) Nutritional programming of gastrointestinal tract development. Is the pig a good model for man? Nutr Res Rev 23, 4–22. 20500926 10.1017/S0954422410000077

[32] Knox MR, Deng K & Nolan JV (2003) Nutritional programming of young sheep to improve later-life production and resistance to nematode parasites: a brief review. Aust J Exp Agric 43, 1431–1435.

[33] Geurden I, Borchert P, Balasubramanian MN, et al (2013) The positive impact of the early-feeding of a plant-based diet on its future acceptance and utilization in rainbow trout. PLOS ONE 8, e83162. 24386155 10.1371/journal.pone.0083162 PMC3873907

[34] Fang L, Liang X-F, Zhou Y, et al (2014) Programming effects of high-carbohydrate feeding of larvae on adult glucose metabolism in zebrafish, *Danio rerio* . Br J Nutr 111, 808–818. 24112146 10.1017/S0007114513003243

[35] Perera E & Yúfera M (2016) Soybean meal and soy protein concentrate in early diet elicit different nutritional programming effects on juvenile zebrafish. Zebrafish 13, 61–69. 26716770 10.1089/zeb.2015.1131

[36] Perera E & Yúfera M (2017) Effects of soybean meal on digestive enzymes activity, expression of inflammation-related genes, and chromatin modifications in marine fish (*Sparus aurata* L.) larvae. Fish Physiol Biochem 43, 563–578. 27807713 10.1007/s10695-016-0310-7

[37] Fjelldal PG, Hansen TJ, Lock E-J, et al (2015) ) Increased dietary phosphorus prevents vertebral deformities in triploid Atlantic salmon (*Salmo salar* L.). Aquacult Nutr 22, 72–90.

[38] Taylor JF, Waagbø R, Diez-Padrisa M, et al (2015) Adult triploid Atlantic salmon (*Salmo salar*) have higher dietary histidine requirements to prevent cataract development in seawater. Aquacult Nutr 21, 18–32.

[39] Burke H, Sacobie CFD, Lall SP, et al (2010) The effect of triploidy on juvenile Atlantic salmon (*Salmo salar*) response to varying levels of dietary phosphorus. Aquaculture 306, 295–301.

[40] Sacobie CFD, Burke H, Lall SP, et al (2015) The effect of dietary energy level on growth and nutrient utilization by juvenile diploid and triploid brook charr, *Salvelinus fontinalis* . Aquacult Nutr 22, 1091–1100.

[41] Johnstone R & Stet RJM (1995) The production of gyogenetic Atlantic salmon, *Salmo salar* L. Theo Appl Genet 90, 819–826. 10.1007/BF00222017 24172924

[42] Fraser TWK, Hansen T, Fleming MS, et al (2015) The prevalence of vertebral deformities is increased with higher egg incubation temperatures and triploidy in Atlantic salmon *Salmo salar* L. J Fish Dis 38, 75–89. 25664364 10.1111/jfd.12206

[43] Association of Official Analytical Chemists (2000) Official Methods of Analysis, 17th ed. Arlington, VA: AOAC International.

[44] Folch J, Lees M & Sloane-Stanley GH (1957) A simple method for the isolation and purification of total lipids from animal tissues. J Biol Chem 226, 497–509. 13428781

[45] Christie WW (2003) Lipid Analysis, 3rd ed. Bridgewater: The Oily Press.

[46] Tocher DR & Harvie DG (1988) Fatty acid compositions of the major phosphoglycerides from fish neural tissues; (*n*-3) and (*n*-6) polyunsaturated fatty-acids in rainbow trout (*Salmo gairdneri*) and cod (*Gadus morhua*) brains and retinas. Fish Physiol Biochem 5, 229–239. 24226784 10.1007/BF01874800

[47] Urán PA, Scharama JW, Rombout JHWM, et al (2008) Soybean meal-induced enteritis in Atlantic salmon (*Salmo salar* L.) at different temperatures. Aquacult Nutr 14, 324–330.

[48] Fournier V, Huelvan C & Desbruyeres E (2004) Incorporation of a mixture of plant feedstuffs as substitute for fish meal in diets of juvenile turbot (*Psetta maxima*). Aquaculture 236, 451–465.

[49] Soltan MA, Hanafy MA & Wafa MIA (2008) Effect of replacing fish meal by a mixture of different plant protein sources in Nile tilapia (*Oreochromis niloticus* L.) diets. Global Vet 2, 157–164.

[50] Pratoomyot J, Bendiksen EA, Bell JG, et al (2010) Effects of increasing replacement of dietary fishmeal with plant protein sources on growth performance and body liquid composition of Atlantic salmon (*Salmo salar* L.). Aquaculture 305, 124–132.

[51] Palace VP & Werner J (2006) Vitamins A and E in the maternal diet influence egg quality and early life stage development in fish: a review. Sci Mar 70S, 41–57.

[52] Izquierdo MS, Fernandez-Palacios H & Tacon AG (2001) Effect of broodstock nutrition on reproductive performance of fish. Aquaculture 197, 25–42.

[53] Zambonino-Infante JL & Cahu CL (2001) Ontogeny of the gastrointestinal tract of marine fish larvae. Comp Biochem Physiol C Toxicol Pharmacol 130, 477–487. 11738635 10.1016/s1532-0456(01)00274-5

[54] McGeachy SA, Benfey TJ & Friars GW (1995) Freshwater performance of triploid Atlantic salmon (*Salmo salar*) in New Brunswick aquaculture. Aquaculture 137, 333–341.

[55] O’Flynn FM, McGeachy SA, Friars GW, et al (1997) Comparisons of cultured triploid and diploid Atlantic salmon (*Salmo salar*). ICES J Mar Sci 54, 1160–1165.

[56] Cotter D, O’Donovan V, Drumm A, et al (2002) Comparison of freshwater and marine performances of all-female diploid and triploid Atlantic salmon (*Salmo salar* L.). Aquacult Res 33, 43–53.

[57] Galbreath PF, St Jean W, Anderson V, et al (1994) Freshwater performance of all-female diploid and triploid Atlantic salmon. Aquaculture 128, 41–49.

[58] Taylor JF, Preston AC, Guy D, et al (2011) Ploidy effects on hatchery survival, deformities, and performance in Atlantic salmon (*Salmo salar*). Aquaculture 315, 61–68.

[59] Smedley MA, Clokie BGJ, Migaud H, et al (2016) Dietary phosphorus and protein supplementation enhances seawater growth and reduces severity of vertebral malformation in triploid Atlantic salmon (*Salmo salar* L.). Aquaculture 451, 357–368.

[60] Sambraus F, Fjelldal PG, Remø SC, et al (2017) Water temperature and dietary histidine affect cataract formation in Atlantic salmon (Salmo salar L.) diploid and triploid yearling smolt. J Fish Dis (Epublication ahead of print version 11 February 2017). 10.1111/jfd.12594 28188652

[61] Glencross BD, Carter CG, Duijster N, et al (2004) A comparison of the digestibility of a range of lupin and soybean protein products when fed to either Atlantic salmon (*Salmo salar*) or rainbow trout (*Oncorhynchus mykiss*). Aquaculture 237, 333–346.

[62] Denstaldi V, Storebakken T, Svilhus B, et al (2007) A comparison of online phytase pre-treatment of vegetable feed ingredients and phytase coating in diets for Atlantic salmon (*Salmo salar* L.) reared in cold water. Aquaculture 269, 414–426.

[63] Hamre K, Sissiner NH, Lock E-J, et al (2016) Antioxidant nutrition in Atlantic salmon (*Salmo salar*) parr and post-smolt, fed diets with high inclusion of plant ingredients and graded levels of micronutrients and selected amino acids. PeerJ 4, e2688. 27843721 10.7717/peerj.2688 PMC5103829

[64] Hemre G-I, Lock E-J, Olsvik PA, et al (2016) Atlantic salmon (*Salmo salar*) require increased dietary levels of B-vitamins when fed diets with high inclusion of plant based ingredients. PeerJ 4, e2493. 27703849 10.7717/peerj.2493 PMC5047143

[65] Balasubramanian MN, Panserat S, Dupont-Nivet M, et al (2016) Molecular pathways associated with the nutritional programming of plant-based diet acceptance in rainbow trout following an early feeding exposure. BMC Genomics 17, 449. 27296167 10.1186/s12864-016-2804-1 PMC4907080

[66] West-Eberhard M-J (1989) Phenotypic plasticity and the origins of diversity. Ann Rev Ecol Syst 20, 249–278.

[67] Nuez-Ortín WG, Carter CG, Wilson R, et al (2017) Triploid Atlantic salmon show similar performance, fatty acid composition and proteome response to diploids during early freshwater rearing. Comp Biochem Physiol D Genomics Preteomics 22, 67–77. 10.1016/j.cbd.2017.02.005 28214702

[68] Small SA & Benfey TJ (1987) Cell size in triploid salmon. J Exp Zool 241, 339–342.

[69] Peruzzi S, Hagen Ø & Jobling M (2015) ) Gut morphology of diploid and triploid Atlantic salmon (*Salmo salar* L.). Aquacult Int 23, 1105–1108.

[70] Tocher DR, Bell JG, Dick JR, et al (1997) Fatty acyl desaturation in isolated hepatocytes from Atlantic salmon (*Salmo salar*): stimulation by dietary borage oil containing ɣ-linolenic acid. Lipids 32, 1237–1247. 9438233 10.1007/s11745-006-0159-0

[71] Torstensen BE, Frøyland L & Lie Ø (2004) Replacing dietary fish oil with increasing levels of rapeseed oil and olive oil – effects on Atlantic salmon (*Salmo salar* L.) tissue and lipoprotein lipid composition and lipogenic enzyme activities. Aquacult Nutr 10, 175–192.

[72] Sanden M, Stubhaug I, Berntssen MHG, et al (2011) Atlantic salmon (*Salmo salar* L.) as a net producer of long-chain marine ω-3 fatty acids. J Agric Food Chem 59, 12697–12706. 22017199 10.1021/jf203289s

[73] Turchini GM, Francis DS, Keast RSJ, et al (2011) Transforming salmonid aquaculture from a consumer to a producer of long chain omega-3 fatty acids. Food Chem 124, 609–614.

[74] Rosenlund G, Torstensen BE, Stubhaug I, et al (2016) Atlantic salmon require long-chain *n*-3 fatty acids for optimal growth throughout the seawater period. J Nutr Sci 5, e19. 27293556 10.1017/jns.2016.10 PMC4891698

[75] Tocher DR (2003) Metabolism and functions of lipids and fatty acids in teleost fish. Rev Fisheries Sci 11, 107–184.

[76] Tocher DR (2010) Fatty acid requirements in ontogeny of marine and freshwater fish. Aquacult Res 41, 717–732.

[77] Glencross BD (2009) Exploring the nutritional demand for essential fatty acids by aquaculture species. Rev Aquacult 1, 71–124.

[78] Leaver MJ, Bautista JM, Björnsson T, et al (2008) Towards fish lipid nutrigenomics: current state and prospects for fin-fish aquaculture. Rev Fisheries Sci 16, Suppl. 1, 71–92.

[79] Vagner M, Robin JH, Zambonino-Infante JL, et al (2009) Ontogenic effects of early feeding of sea bass (*Dicentrarchus labrax*) larvae with a range of dietary *n*-3 highly unsaturated fatty acid levels on the functioning of polyunsaturated fatty acid desaturation pathways. Br J Nutr 101, 1452–1462. 18838020 10.1017/S0007114508088053

[80] Bell MV & Tocher DR (2009) Biosynthesis of fatty acids; general principles and new directions In Lipids in Aquatic Ecosystems, pp. 211–236 [MT Arts, M Brett and M Kainz, editors]. New York: Springer.

[81] Tocher DR (2015) Omega-3 long-chain polyunsaturated fatty acids and aquaculture in perspective. Aquaculture 449, 94–107.

[82] Bell JG, McEvoy J, Tocher DR, et al (2001) Replacement of fish oil with rapeseed oil in diets of Atlantic salmon (*Salmo salar*) affects tissue lipid compositions and hepatocyte fatty acid metabolism. J Nutr 131, 1535–1543. 11340112 10.1093/jn/131.5.1535

[83] Bell JG, Henderson RJ, Tocher DR, et al (2002) Substituting fish oil with crude palm oil in the diet of Atlantic salmon (*Salmo salar*) affects muscle fatty acid composition and hepatic fatty acid metabolism. J Nutr 132, 222–230. 11823582 10.1093/jn/132.2.222

[84] Bell JG, Pratoomyot J, Strachan F, et al (2010) Growth, flesh adiposity and fatty acid composition of Atlantic salmon (*Salmo salar*) families with contrasting flesh adiposity: effects of replacement of dietary fish oil with vegetable oils. Aquaculture 306, 225–232.

[85] Torstensen BE, Espe M, Sanden M, et al (2008) Novel production of Atlantic salmon (*Salmo salar*) protein based on combined replacement of fish meal and fish oil with plant meal and vegetable oil blends. Aquaculture 285, 193–200.

[86] Turchini GM, Ng W-K & Tocher DR (editors) (2010) Fish Oil Replacement and Alternative Lipid Sources in Aquaculture Feeds. Boca Raton, FL: Taylor & Francis, CRC Press.

[87] Torstensen BE & Tocher DR (2010) The effects of fish oil replacement on lipid metabolism of fish In Fish Oil Replacement and Alternative Lipid Sources in Aquaculture Feeds, pp. 405–437 [GM Turchini, W-K Ng and DR Tocher, editors]. Boca Raton, FL: Taylor & Francis, CRC Press.

[88] Krogdahl Å, Bakke-McKellep AM & Bæverfjord G (2003) Effects of graded levels of standard soybean meal on intestinal structure, mucosal enzyme activities, and pancreatic response in Atlantic salmon (*Salmo salar*). Aquacult Nutr 9, 361–371.

[89] Hedrera MI, Galdames JA, Jimenez-Reyes M, et al (2013) Soybean meal induces intestinal inflammation in Zebrafish Larvae. PLOS ONE 8, e69983. 23894568 10.1371/journal.pone.0069983 PMC3720926

[90] Morais S, Pratoomyot J, Taggart JB, et al (2011) Genotype-specific responses in Atlantic salmon (*Salmo salar*) subject to dietary fish oil replacement by vegetable oil: a liver transcriptomic analysis. BMC Genomics 12, 255. 21599965 10.1186/1471-2164-12-255 PMC3113789

[91] Tacchi L, Secombes CJ, Bickerdike R, et al (2012) Transcriptomic and physiological responses to fishmeal substitution with plant proteins in formulated feed in farmed Atlantic salmon (*Salmo salar*). BMC Genomics 13, 363. 22853566 10.1186/1471-2164-13-363 PMC3526460

